# Research on the regulation of gut microbiota homeostasis and immune function in asthmatic mice by Huanglong Zhixiao Formula

**DOI:** 10.3389/fmicb.2025.1726388

**Published:** 2026-01-05

**Authors:** Yong-xia Chen, Yin-shuang Xuan, Ming-hang Wang, Ya Li, Sheng-ming Shi, Hao-yu Zhao, Yi-hao Niu, Min Chen, Su-yun Li

**Affiliations:** 1 Faculty of Chinese Medicine and State Key Laboratory of Quality Research in Chinese Medicines, Macau University of Science and Technology, Taipa, Macao SAR, China; 2 Department of Respiratory and Critical Care Medicine, Chinese Medicine Pharmacology (Respiratory) Laboratory, The First Affiliated Hospital of Henan University of Chinese Medicine, Zhengzhou, Henan, China; 3 MUST Science and Technology, Innovation Technology Research Institute of Guangdong-Macao In-Depth Cooperation Zone in Hengqin, Hengqin, China

**Keywords:** asthma, gut microbiota, Huanglong Zhixiao Formula, inflammation, intestinal barrier function

## Abstract

**Background:**

Asthma affects approximately 334 million people worldwide. Accumulating evidence indicates that gut dysbiosis exacerbates airway inflammation through the gut–lung axis. In the present study, using an OVA-induced murine model of asthma, we investigated whether Huanglong Zhixiao Formula (HLZXF) restores gut lung homeostasis by reshaping the gut microbiota and enhancing intestinal barrier function, thereby attenuating pulmonary pathological changes.

**Methods:**

Female BALB/c mice were randomly assigned to three groups (*n* = 15 per group): Control (C), Asthma Model (MX), and HLZXF-treated (ZG) groups. Asthma was induced by OVA sensitization and challenge over a 6-week period. The ZG group received daily oral gavage of HLZXF, 1 h prior to each OVA challenge. Fecal samples were collected for metagenomic sequencing. Lung and intestinal tissues were excised for HE and IHC staining of tight junction proteins, including Claudin, Occludin, and ZO-1. Alpha and beta diversity analyses were conducted to evaluate the composition and structure of the gut microbiota.

**Results:**

We analyzed the structure of the gut microbiota, detected the expression levels of intestinal barrier-related proteins, and assessed inflammatory injury in the lungs and intestines. Results demonstrated that HLZXF significantly ameliorated gut microbiota dysbiosis in asthmatic mice, as evidenced by the significant enrichment of *Heminiphilus faecis* and *Paramuribaculum intestinale*. Additionally, certain fungal taxa, such as *Piromyces finnis* and *Rhizopus arrhizus*, were significantly enriched in the ZG group. HLZXF also significantly upregulated the expression levels of the tight junction proteins Claudin, Occludin, and ZO-1 in intestinal tissues, thereby promoting the repair of the intestinal mucosal barrier. Furthermore, HLZXF significantly attenuated inflammatory cell infiltration and tissue injury in the lungs and intestines, alleviated alveolar septal thickening, and enhanced the integrity of the intestinal mucosal barrier.

**Conclusion:**

This study elucidates the potential therapeutic mechanisms of HLZXF in the treatment of asthma from the perspective of gut microbiota and intestinal barrier function. It highlights that HLZXF can attenuate pulmonary inflammation by regulating the balance of gut microbiota and enhancing intestinal barrier function.

## 1 Introduction

Asthma is a common chronic airway inflammatory disease affecting approximately 334 million individuals globally (Mosnaim et al., 2025). In terms of pathology, its main features are characterized by airway inflammation, airway remodeling, and airway hyperresponsiveness. The prevalence of asthma varies substantially among regions and populations. Developed countries typically exhibit higher asthma prevalence, whereas that in developing countries is relatively lower. However, amid the acceleration of urbanization and the worsening of environmental pollution, the prevalence of asthma in developing countries has been on the rise in recent years (Asher et al., 2006; To et al., 2012; Papi et al., 2018; Mortimer et al., 2022). Children and adolescents are high-risk groups for asthma, although the incidence among adults also remains considerable. Gender also influences asthma incidence: during childhood, the incidence is higher in males than in females, whereas in adulthood, it is higher in females than in males (Leynaert et al., 2012; Andersson et al., 2013).

The pathophysiological mechanisms underlying asthma are complex, involving the interplay between diverse cell types and inflammatory mediators. These key cells (eosinophils, mast cells, T lymphocytes, macrophages, and airway epithelial cells) interact synergistically and release numerous inflammatory mediators, such as histamine, leukotrienes, cytokines, and chemokines, thereby triggering inflammatory responses in the airways (Simpson et al., 2006; Hallstrand et al., 2018; Chan et al., 2019; Elieh Ali Komi and Bjermer, 2019; Camoretti-Mercado and Lockey, 2021). Actions of these inflammatory mediators lead to contraction of airway smooth muscle, inducing airway narrowing, while simultaneously stimulating enhanced mucus gland secretion, which in turn leads to mucus accumulation within the airways (Carter and Bradding, 2011; Elieh Ali Komi and Bjermer, 2019). Furthermore, inflammation causes structural damage to airway epithelial cells, impairing the structural integrity of the airway epithelium and further exacerbating airway hyperresponsiveness (Puddicombe et al., 2000; Comhair et al., 2005). This heightened hyperresponsiveness renders the airways overly reactive to diverse stimuli (e.g., cold air, allergens, exercise, or chemical irritants), triggering excessive bronchoconstriction and subsequent symptoms including wheezing, coughing, and dyspnea. As inflammation persists, patients with asthma may also develop airway remodeling: a progressive, irreversible pathological change characterized by airway smooth muscle hyperplasia, thickening of the basement membrane, collagen deposition, and mucus gland hypertrophy (Boulet, 2018; Tliba and Panettieri, 2019; Banno et al., 2020). These structural alterations further exacerbate airway narrowing, diminish airway reversibility, and represent a primary factor contributing to the refractory nature of asthma.

With the growing depth of microbiomics research in recent years, the association between asthma and the gut microbiota has emerged as a key research focus. The gut microbiota—defined as the collective term for microorganisms residing in the human gut—constitutes an ecosystem encompassing diverse microorganisms, including bacteria and fungi. Through interactions with the immune, digestive, and nervous systems, it exerts a profound influence on human health (Kau et al., 2011; Pflughoeft and Versalovic, 2012; Shreiner et al., 2015). A growing body of evidence indicates that asthmatic patients frequently exhibit alterations in the composition and function of the gut microbiota, which may modulate the development and progression of asthma via multiple mechanisms (Depner et al., 2020; Patrick et al., 2020; Li et al., 2021). In healthy individuals, the gut microbiota maintains a dynamic equilibrium with the host, a state critical for preserving gut barrier integrity, regulating immune function, and facilitating nutrient absorption (Kau et al., 2011; Pflughoeft and Versalovic, 2012; Shreiner et al., 2015). In asthmatic patients, the gut bacterial composition differs significantly, typically marked by a reduction in beneficial bacterial taxa and an expansion of pathogenic or pro-inflammatory bacteria. Analysis of fecal samples from children reveals that those with higher relative abundances of *Streptococcus* and *Bacteroides* and lower relative abundances of *Bifidobacterium*, *Lachnospira*, *Veillonella*, *Faecalibacterium*, *Akkermansia*, and *Rothia* exhibit an elevated risk of asthma development. Furthermore, transplantation of representative strains from the genera *Lachnospira*, *Veillonella*, *Faecalibacterium*, and *Rothia* into mouse models has been shown to attenuate airway inflammation (Arrieta et al., 2015; Fujimura et al., 2016; Auchtung et al., 2018). In addition, a strong association exists between gut fungal communities and asthma: gut fungi can modulate pulmonary immune function via the gut-lung axis, thereby impacting asthma pathogenesis (Wu et al., 2021). The gut microbiota shapes the pulmonary immune microenvironment via signaling cascades along the gut-lung axis (Frati et al., 2018). Gut microbiota dysbiosis induces aberrant activation and migration of inflammatory cells, which then traffic to the lungs, secrete inflammatory mediators, and thereby exacerbate airway inflammation (Adcock et al., 2008; Essilfie et al., 2012; Wu et al., 2021), gut microbiota-derived metabolites can reach the lungs via the systemic circulation, regulate the activity of pulmonary immune cells, and modulate airway inflammation pathogenesis (Trompette et al., 2014; Cait et al., 2018; Wu et al., 2021).

Glucocorticoids and anticholinergic agents are commonly used in the treatment of asthma. While they can effectively control asthma symptoms, they may induce a series of adverse effects (Busse et al., 2021; Casale et al., 2022). Long-term or high-dose use of glucocorticoids can cause metabolic disorders, inhibit immune function, and increase the risk of infections (Howell et al., 2024). Frequent use of anticholinergic agents can slow intestinal peristalsis, leading to constipation, and may also cause adverse effects such as urinary retention (Casale et al., 2022). Traditional Chinese Medicine (TCM) boasts a long-standing history and extensive clinical experience in asthma management, and its unique theoretical framework and therapeutic approaches offer diverse treatment options for individuals with asthma (Li and Brown, 2009). From the TCM perspective, the core therapeutic principle of TCM for asthma is “treating both symptoms and root causes,” with the goals of alleviating symptoms, reducing attack frequency, and enhancing patients’ quality of life by regulating the body’s overall functional status. In clinical practice, TCM treatments for asthma primarily encompass traditional Chinese herbal decoctions, acupuncture, tuina (Chinese massage), and acupoint application.

Huanglong Zhixiao Formula (HLZXF) is a multi-herb formula in TCM, characterized by its effects of clearing heat, resolving phlegm, relieving cough, and alleviating asthma. Its main components include *Honey-fried Ephedra* Herba (*Ephedra sinica* Stapf, processed by honey-frying), *Belamcanda chinensis* (L.) DC., *Pheretima aspergillum* (Perrier), *Peucedanum praeruptorum* Dunn, Stir-fried Perillae Fructus (*Perilla frutescens* (L.) Britt., processed by stir-frying), *Fritillaria thunbergii* Miq., Spina Gleditsiae (*Gleditsia sinensis* Lam.), Roasted Mori Cortex (*Morus alba* L., processed by roasting), Stir-fried Ginkgo Semen (*Ginkgo biloba* L., processed by stir-frying), *Prunus mume* (Siebold & Zucc.), and *Glycyrrhiza uralensis* Fisch. *Belamcandae Rhizoma* and *Ephedra Herba* are the primary components of Shegan Mahuang Decoction—a classic TCM formula derived from Jin Kui Yao Lue (Golden Chamber Synopsis), Volume 1, renowned for its effects of relieving lung congestion, resolving phlegm and relieving cough (Lin et al., 2020). *Ephedra sinica*—one of HLZXF’s components—contains active ingredients with anti-inflammatory properties that can enhance immune function, thereby alleviating asthma-related symptoms (Zhuo et al., 2024). However, the pharmacological effects and underlying mechanisms of HLZXF in asthma treatment remain unclear. This study aims to investigate the regulatory effects of HLZXF on gut microbiota homeostasis in asthmatic mice, as well as its potential immunological mechanisms, with the goal of providing a new theoretical basis and therapeutic targets for asthma treatment.

## 2 Materials and methods

### 2.1 Drugs and reagents

HLZXF was kindly provided by the First Affiliated Hospital of Henan University of Chinese Medicine. The main components include: *Honey-fried Ephedra* Herba (*Ephedra sinica* Stapf, processed by honey-frying):120 g, *Belamcanda chinensis* (L.) DC: 200 g., *Pheretima aspergillum* (Perrier): 200 g, *Peucedanum praeruptorum* Dunn: 240 g, Stir-fried Perillae Fructus (*Perilla frutescens* (L.) Britt., processed by stir-frying): 200 g, *Fritillaria thunbergii* Miq: 180 g., Spina Gleditsiae (*Gleditsia sinensis* Lam.):180 g, Roasted Mori Cortex (*Morus alba* L., processed by roasting): 400 g, Stir-fried Ginkgo Semen (*Ginkgo biloba* L., processed by stir-frying): 200 g, *Prunus mume* (Siebold & Zucc.): 200 g, and *Glycyrrhiza uralensis* Fisch: 120 g. The formula was prepared as follows: first, A portion of Fritillaria thunbergii Miq. is ground into fine powder and sieved. The remaining Fritillaria thunbergii Miq. is decocted with other materials in water twice, and the two decoctions are combined, the herbs were decocted, the decoction was filtered, and the filtrate was concentrated to a clear extract with a relative density of 1.10–1.20. An appropriate amount of dextrin was added, the mixture was dried and pulverized, and then mixed with the aforementioned Fritillaria thunbergii powder; additional dextrin was added as required. After further drying, the mixture was formulated into a powdered form; prior to use, it was reconstituted to the desired concentration with normal saline. Ovalbumin (OVA; batch no: SLBF0342V; Sigma-Aldrich, Darmstadt, Germany) was used for the sensitization and challenge of mice, while aluminum hydroxide gel (Alum; batch no: XF342045; Thermo Fisher Scientific Inc., Waltham, Massachusetts, USA) was used as an adjuvant. Antibodies: ZO-1 antibody (cat. no: 115686), Occludin antibody (cat. no: 111401), and Claudin antibody (cat. no: 152543); all antibodies were purchased from Wuhan Servicebio Technology Co., Ltd. (Wuhan, China).

### 2.2 Experimental animals and grouping

Female BALB/c mice (6–8 weeks old, specific pathogen-free (SPF) grade, body weight 20 ± 2 g) were purchased from Beijing SPF Biotechnology Co., Ltd. (Beijing, China). The mice were housed in a controlled environment maintained at 22 ± 2 °C, 50% ± 10% relative humidity, and a 12-h light/12-h dark cycle, with ad libitum access to standard chow and water. Following a 1-week acclimatization period, the experiment was initiated. Furthermore, to eliminate the impact of circadian fluctuations in estrogen levels, all animal treatments and sample collections were conducted at the same time each day to avoid systematic errors induced by circadian variation. This study was approved by the Animal Ethics Committee of the First Affiliated Hospital of Henan University of Chinese Medicine (Approval No: YFYDW2023002).

Mice were randomly assigned to three groups (*n* = 15 per group) using the RAND() function: Control (C) group, Asthma Model (MX) group, and HLZXF-treated (ZG) group. An asthma mouse model was established via sensitization and challenge with ovalbumin (OVA) and aluminum hydroxide gel (Al(OH)3). On days 0, 7, and 14, mice in the MX and ZG groups received an intraperitoneal injection of a sensitizing solution (0.2 mL) containing 100 μg OVA and 2 mg Al(OH)3 gel, while mice in the C group received an equivalent volume of normal saline via intraperitoneal injection.

From day 21 onwards, all mice were placed in a nebulization chamber: mice in the MX and ZG groups were nebulized with 2.5% OVA, while those in the C group were nebulized with an equivalent volume of normal saline. This nebulization procedure was performed three times per week, with each session lasting 30 min, for a total of 6 weeks. Concurrently, mice in the ZG group were administered the formula via gavage at a dose of 14.56 g/kg/day, 1 h prior to each nebulization challenge; mice in the C and MX groups received an equivalent volume of normal saline via gavage at the same time point. This gavage treatment was continued for 6 weeks. The dose of HLZXF administered to mice was calculated using an equivalent dose conversion formula: D(mouse) = D(human) × (HI(mouse)/HI(human)) × (W(mouse)/W(human))^(2/3), where D = dose, HI = body size coefficient, and W = body weight.

### 2.3 Tissue sample collection and processing

#### 2.3.1 Lung tissue sample collection

After the experiment, the right lung tissue of mice was rapidly harvested, and blood stains and contaminants were removed. Subsequently, the surrounding adherent tissues connected to the lung tissue were completely dissected, and the lung tissue was rinsed repeatedly with normal saline three times. A portion of the tissue was fixed in 10% neutral buffered formalin for 72 h, followed by paraffin embedding and routine sectioning at a thickness of 4 μm. These sections were used for hematoxylin and eosin (H&E) staining to observe pathological changes in the lung tissue.

#### 2.3.2 Intestinal tissue sampling

Segments of the entire small intestine and large intestine from mice were excised. Intestinal contents were removed, and the intestinal segments were then rinsed twice with normal saline. The intestinal tissues were then fixed in freshly prepared 10% NBF for 72 h. The fixed tissues were then embedded in paraffin wax, and 4-μm-thick sections were prepared from the embedded blocks. Routine H&E staining was performed to evaluate histomorphological alterations in the colonic tissues.

#### 2.3.3 Fecal sample collection

A small incision was made at the distal end of the cecum, and the cecal contents were gently squeezed into sterile 2 mL EP tubes. The samples were immediately flash-frozen in liquid nitrogen and subsequently transferred to a − 80 °C freezer for long-term storage. These samples were used for metagenomic sequencing to assess compositional and quantitative changes in the gut microbiota.

#### 2.3.4 Histopathological examination

Following fixation in 10% NBF, which is consistent with prior protocols, lung and intestinal tissue samples were processed via standard histological procedures: dehydration, clearing, paraffin infiltration, and embedding. From the embedded paraffin blocks, 4-μm-thick sections were prepared using a microtome. Subsequently, the sections were stained with H&E using standard protocols, and the histomorphological features of the lung and intestinal tissues were observed under a light microscope.

#### 2.3.5 Immunohistochemical analysis

Paraffin sections were deparaffinized and rehydrated via sequential immersion in eco-friendly dewaxing solutions (Solution I, Solution II, Solution III; 10 min per immersion) and anhydrous ethanol (Ethanol I, Ethanol II, Ethanol III; 5 min per immersion), followed by rinsing with distilled water. The slides were then washed with PBS (Servicebio, Cat. No: G0002) three times, with each wash lasting 5 min.

Antigen retrieval was conducted using EDTA buffer (pH 8.0; Servicebio, Cat. No: G1206) via microwave heating: the buffer was heated at medium power for 8 min, followed by a 7-min pause, and then further heated at low-medium power for 7 min. After the sections were naturally cooled, they were washed with PBS three times, with each wash lasting 5 min. Endogenous peroxidase activity was inhibited using 3% hydrogen peroxide (H₂O₂; Anjie Gaoke) at room temperature for 25 min in the dark, followed by three washes with PBS, each lasting 5 min. Non-specific binding was blocked using 3% bovine serum albumin (BSA; Servicebio, Cat. No: GC305010) at room temperature for 30 min.

After removing the blocking solution, the sections were incubated overnight at 4 °C with primary antibodies: rabbit anti-ZO-1 (Servicebio, Cat. No: GB115686), rabbit anti-Occludin (Servicebio, Cat. No: GB111401), and mouse anti-Claudin-1 (Servicebio, Cat. No: GB12032); all primary antibodies were diluted at a ratio of 1:500. On the following day, the sections were washed three times with PBS, with each wash lasting 5 min, and then incubated with horseradish peroxidase (HRP)-labeled secondary antibodies (Servicebio) at room temperature for 50 min: goat anti-rabbit IgG (Cat. No: GB23303) and goat anti-mouse IgG (Cat. No: GB23301), both diluted at a ratio of 1:200. After three additional washes with PBS (each lasting 5 min), 3,3′-diaminobenzidine (DAB) chromogen solution (Servicebio, Cat. No.: G1212) was added to the sections, and color development was monitored under a light microscope. The color development reaction was terminated by rinsing the sections with tap water.

The sections were counterstained with hematoxylin solution (Servicebio, Cat. No: G1004) for 3 min, differentiated in differentiation solution (Servicebio, Cat. No: G1039) for 3–5 s, and blued in bluing solution (Servicebio, Cat. No: G1040) for 1–2 min, followed by rinsing with running water. The sections were dehydrated sequentially in 75% ethanol, 85% ethanol, absolute ethanol I, and absolute ethanol II (5 min per immersion), followed by immersion in n-butanol for 5 min, and finally immersion in xylene I for 5 min. After brief air drying at room temperature, the sections were mounted with ultra-clean fast-drying mounting medium (Servicebio, Cat. No: G1404; volume: 100 mL) and observed under a Nikon E100 light microscope for image acquisition.

### 2.4 Gut microbiota analysis

Mouse fecal samples stored at −80 °C were sent to Majorbio Bio-Pharm Technology Co., Ltd. (Shanghai, China) for metagenomic sequencing. Alpha (*α*) diversity and beta (*β*) diversity were analyzed to evaluate significant differences in microbial community composition and structural diversity among groups. Differential taxon identification among groups was performed using the Linear Discriminant Analysis Effect Size (LEfSe) method in R software. LEfSe analysis employed Kruskal-Wallis analysis of variance (ANOVA) and Wilcoxon rank-sum tests (significance level α = 0.05) to identify significantly different taxa, with linear discriminant analysis (LDA) scores ≥ 4 indicating the magnitude of the effect. Additionally, MetaStat (implemented in R) was used to assess differential abundance across six taxonomic levels: phylum, class, order, family, genus, and species, with a significance threshold of *p* < 0.05.

### 2.5 Detection of inflammatory factors

Total RNA was extracted from colon tissues using the TRIzol method. RNA was reverse-transcribed into cDNA in strict accordance with the operating instructions of the kit (Tiangen Biochemical Technology Co., Ltd.; Beijing, China). Quantitative real-time polymerase chain reaction (qPCR) was performed with cDNA as the template. The primers used were as follows: mIL33 (Forward: ATTTCCCCGGCAAAGTTCAG; Reverse: AACGGAGTCTCATGCAGTAGA), mIL22 (Forward: ATGAGTTTTTCCCTTATGGGGAC; Reverse: GCTGGAAGTTGGACACCTCAA), and mGAPDH (Forward: TGTGTCCGTCGTGGATCTGA; Reverse: TTGCTGTTGAAGTCGCAGGAG). The qPCR assay was conducted on the LightCycler^®^ 480 II instrument (Roche Diagnostics Products (Shanghai) Co., Ltd.; Shanghai, China) following the manufacturer’s protocol of the Universal Blue SYBR Green qPCR Master Mix kit (Wuhan Servicebio Technology Co., Ltd.; Wuhan, China). Glyceraldehyde-3-phosphate dehydrogenase (GAPDH) was used as the internal reference gene, and the relative expression levels of target genes were calculated using the 2^-ΔΔCt^ method.

### 2.6 Data analysis

All data were analyzed using R software (version 4.5.0) and presented as mean ± standard deviation (Mean ± SD). Multivariate analysis of variance (ANOVA) was used for comparisons among multiple groups, followed by post-hoc LSD-t tests for pairwise comparisons between groups. Statistical significance was defined as *p < 0.05 and **p < 0.01.

## 3 Results

### 3.1 Establishment of the asthmatic airway model

**Figure 1:**
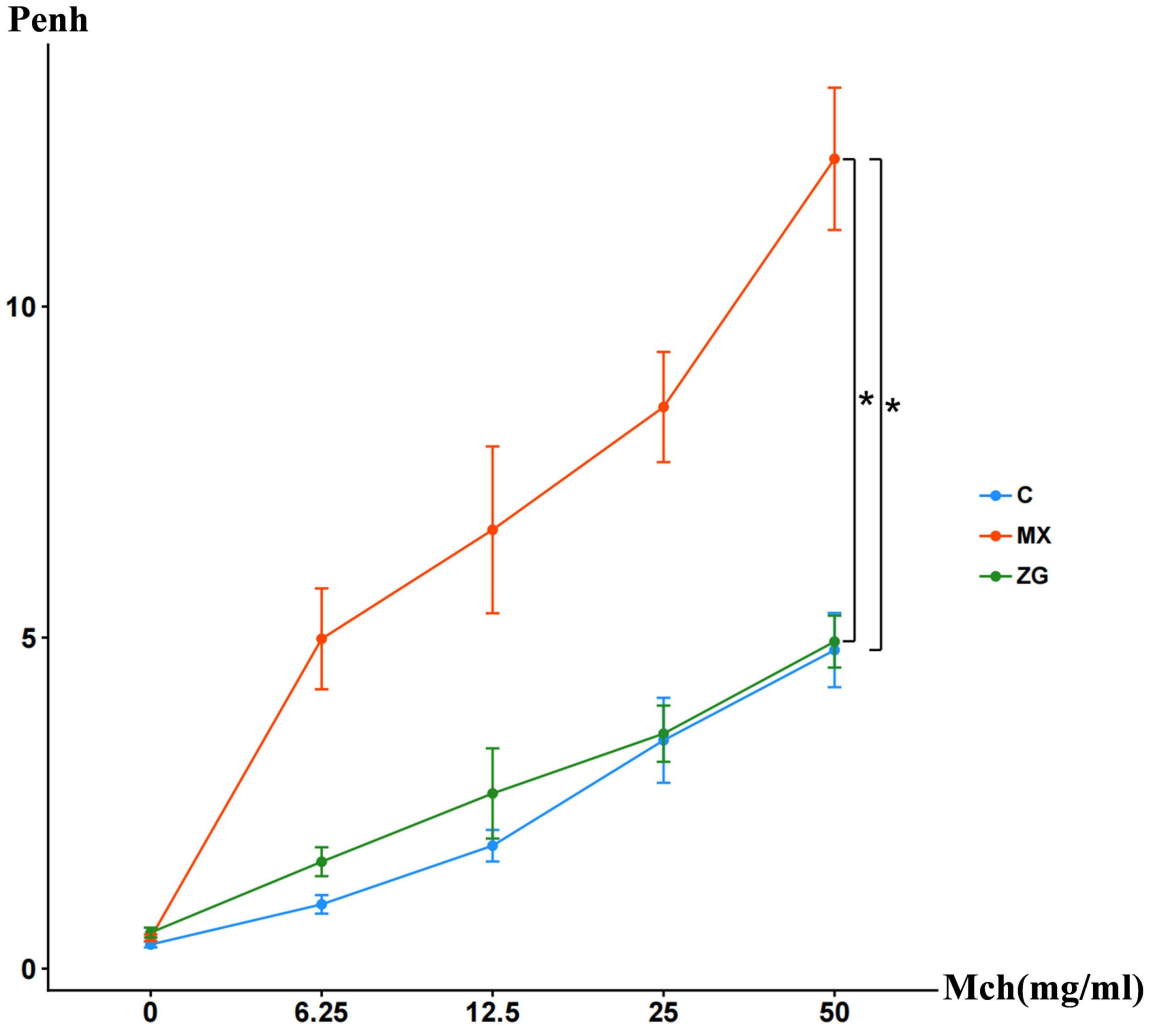
Effects of different concentrations of methacholine (Mch) on Penh values. (C: Control group, MX: Model group, ZG: Huanglong Zhixiao formula group, *p < 0.05).

When nebulized with methacholine (Mch) at a concentration of 25–50 mg/mL, mice in the C group exhibited increased respiratory rate, abdominal breathing, accompanied by salivation, as well as cyanosis of the lips and claws, which are indicative of dyspnea. In contrast to Group C, mice in the MX group developed dyspneic symptoms following nebulization with Mch at a lower concentration of 6.25–12.5 mg/mL. As shown in Figure 1, the Penh value of mice in Group MX was significantly higher than that in the Huanglong Zhixiao Formula intervention group (*p* < 0.05), and also significantly altered compared with the normal control group (*p* < 0.05). These results indicate that the asthmatic airway model was successfully established.

### 3.2 Alpha diversity analysis

**Figure 2:**
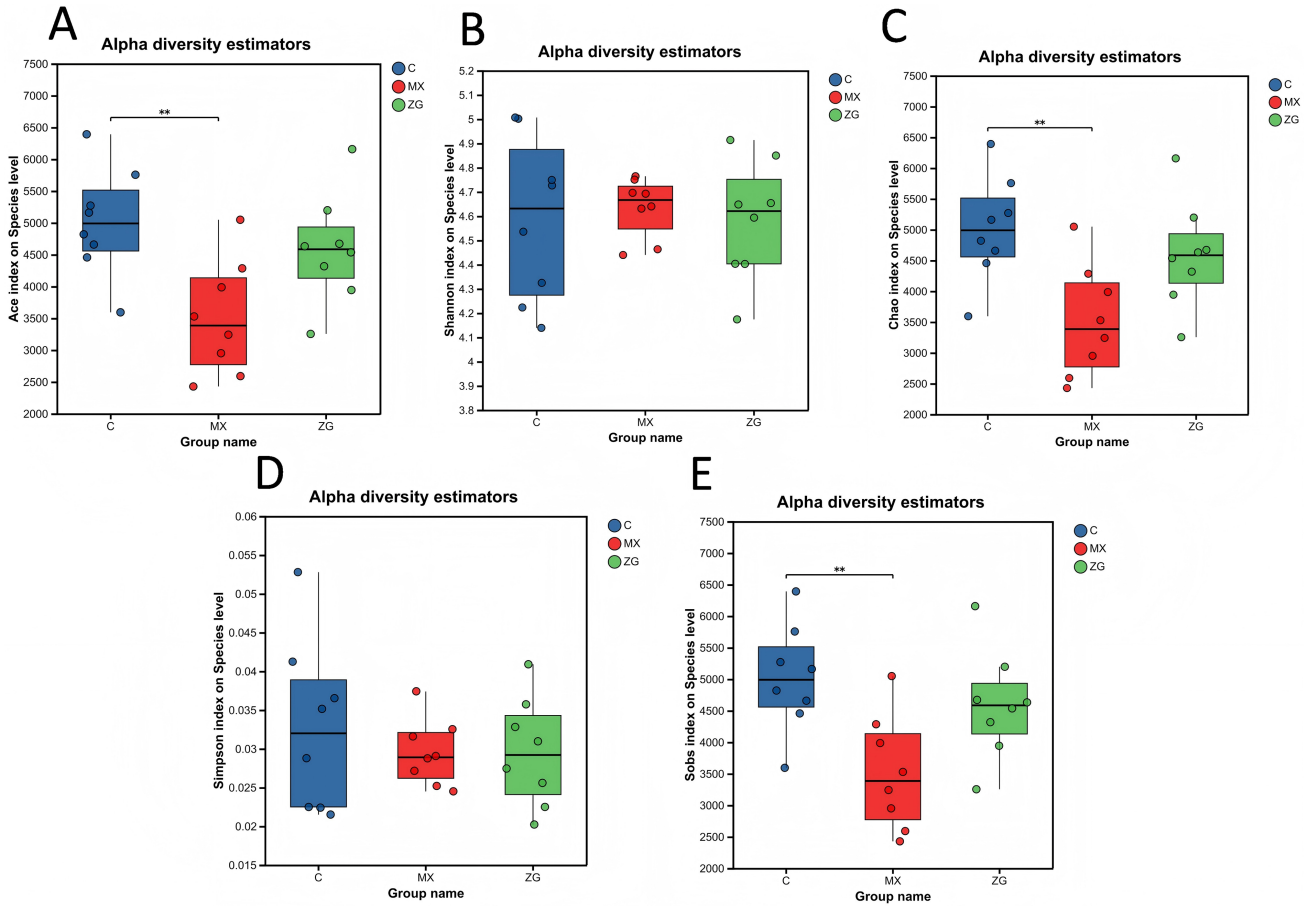
Analysis of *α*-diversity differences among groups. **(A)** ACE index difference analysis; **(B)** Shannon index difference analysis; **(C)** Chao index difference analysis; **(D)** Simpson index difference analysis; **(E)** Sobs index difference analysis. (C: Control group, MX: model group, ZG: Huanglong Zhixiao formula group, **p < 0.01).

Alpha diversity (α diversity) is used to measure the species richness and community diversity within a single sample. Commonly used metrics include the Chao1, Ace, Shannon, Sobs, and Simpson indices. Among these metrics, the Chao1, Sobs, and Ace indices are primarily used to estimate the species richness (i.e., the number of species) in a sample, while the Shannon and Simpson indices are used to assess both species richness and community evenness. To investigate whether HLZXF intervention affects gut microbiota diversity, this study conducted an in-depth evaluation of gut microbiota in three groups of mice: the C group, the MX group, and the ZG group. The results of α diversity analysis showed that, compared with the C group, gut microbiota diversity in the MX group was significantly decreased. However, no significant difference was observed in gut microbiota diversity between the ZG group and the C group. Specifically, for the Ace, Chao1, and Sobs indices, the C group showed significant differences compared to the MX group (*p* < 0.01), while no significant differences were found between the C group and the ZG group. This result indicates that HLZXF intervention restored gut microbiota abundance compared to the MX group. For the Shannon and Simpson indices, no significant differences were observed among the three groups (C, MX, and ZG groups). This finding suggests that HLZXF intervention did not affect gut microbiota species richness or community evenness (Figure 2).

### 3.3 Beta diversity analysis

**Figure 3:**
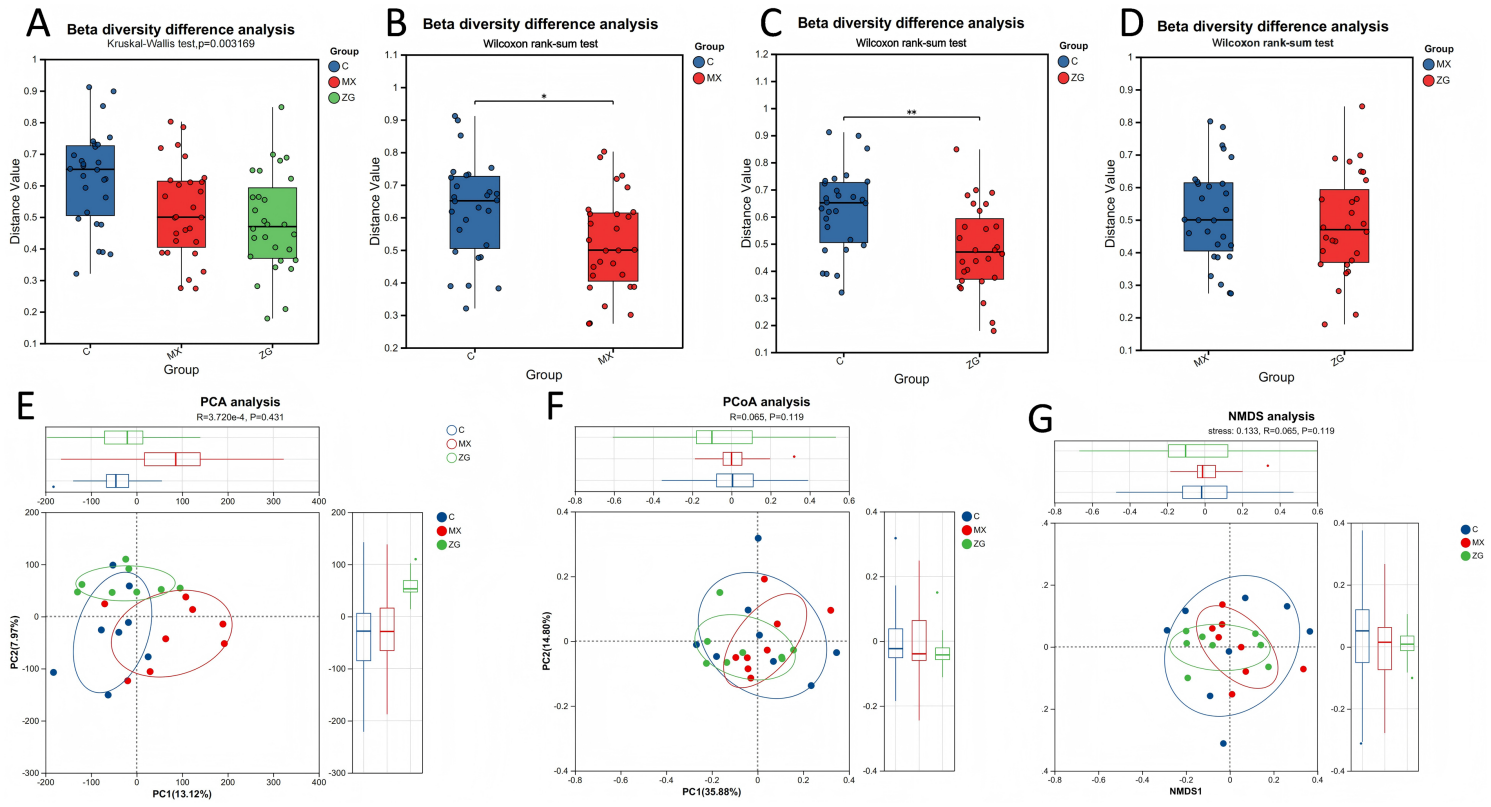
**(A–D)** Analysis of *β*-diversity at the species level among the C, MX, and ZG groups; between the C and MX groups; between the C and ZG groups; and between the MX and ZG groups. **(E)** PCA plot. **(F)** PCoA based on Bray-Curtis distance. **(G)** NMDS analysis. (C: Control group; MX: Model group; ZG: HLZXF group; *p < 0.05, **p < 0.01).

Beta diversity (β diversity) is used to measure differences in species composition between different samples or microbial communities, which reflects the similarity or dissimilarity between them. Commonly used analytical methods for β diversity include Principal Component Analysis (PCA), Principal Coordinates Analysis (PCoA), and Non-metric Multidimensional Scaling (NMDS). Based on the absolute abundance and taxonomic annotation information of Operational Taxonomic Units (OTUs), differences in species composition at the phylum and genus levels were analyzed across all sample groups. Beta diversity analysis results revealed that, compared with the C group, both the MX group and the ZG group exhibited significant changes in gut microbiota species composition, with the ZG group showing more pronounced alterations. Specifically, compared to the C group, the MX group showed significant changes in gut microbiota composition (*p* < 0.05), while the ZG group (HLZXF-treated group) exhibited even more pronounced differences in gut microbiota species composition (*p* < 0.01). This result indicates that HLZXF intervention significantly affects the species composition of the gut microbiota (Figure 3).

### 3.4 Bacterial composition and differential bacterial analysis

**Figure 4:**
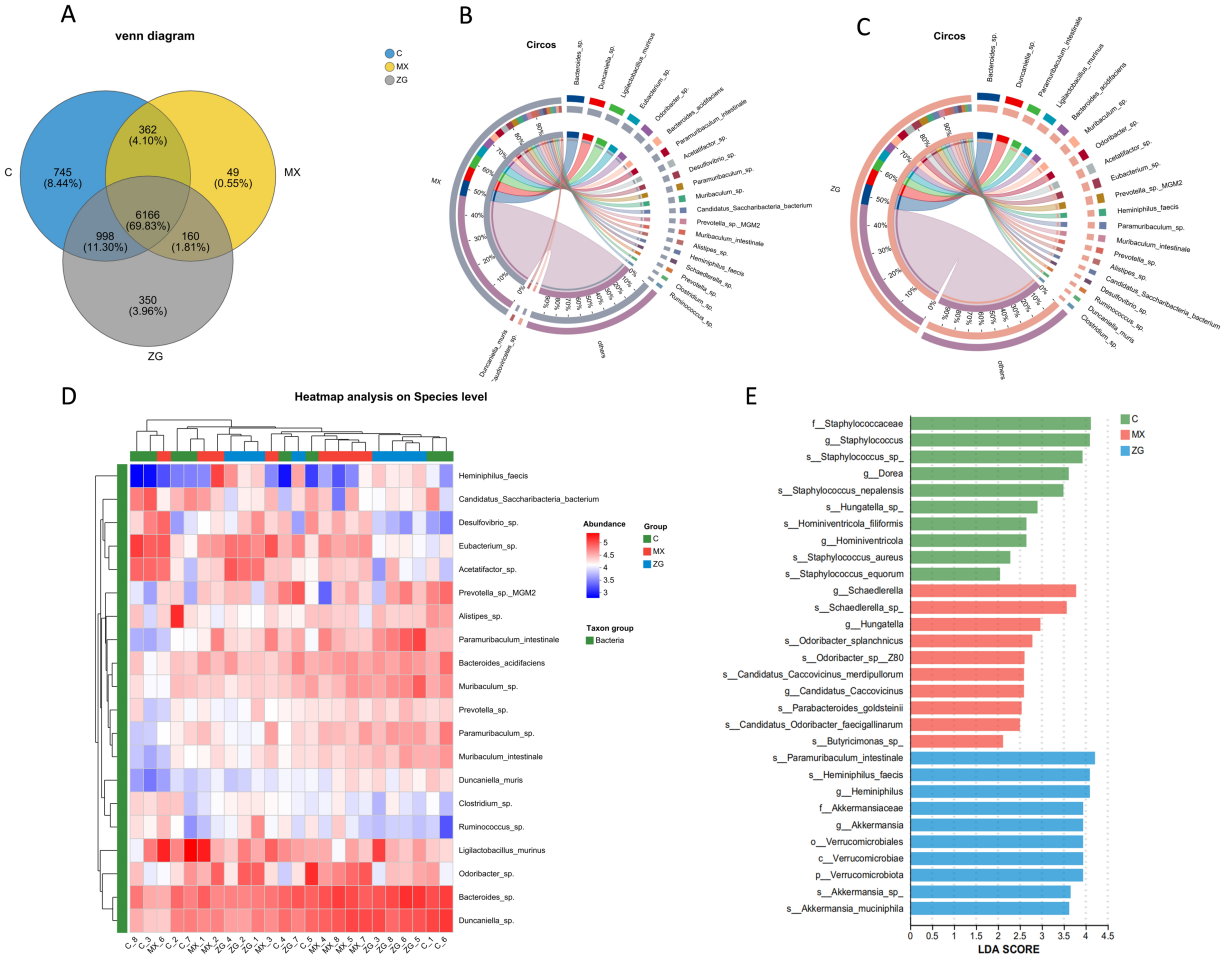
Bacterial composition and differential bacterial analysis in each group. **(A)** Venn diagram of OTU levels across different sample groups; **(B,C)** Circos plot of bacterial at the species level in the MX/ZG group (The left semicircle represents the species abundance composition of samples, with different colors indicating different species and the length corresponding to the abundance proportion. The right semicircle shows the distribution proportion of species among samples, with colors representing samples and the length corresponding to the proportion. The width of the connecting bands indicates the abundance or distribution proportion of species, and the values outside the circle represent the abundance of species); **(D)** Heatmap analysis of bacterial at the species level for each group (only the top 20 are displayed); **(E)** LDA (linear discriminant analysis) distribution histogram of bacterial (C: Control group, MX: Model group, ZG: HLZXF group).

Venn diagram analysis was performed based on OTU abundance information. The number of unique OTUs in the three sample groups were 745 in Group C, 49 in Group MX, and 350 in Group ZG, respectively. Additionally, there were 6,166 OTUs that were shared among all three groups (Figure 4A).

After sequence alignment and analysis, the top 10 most abundant genera detected in the ZG group at the genus level were *Bacteroides*, *Duncaniella*, *Muribaculum*, *Odoribacter*, *Acetatifactor*, *Eubacterium*, *Prevotella*, *Paramuribaculum*, *Alistipes*, and *Desulfovibrio*. At the species level, the most abundant species included *Paramuribaculum intestinale*, *Ligilactobacillus murinus*, *Bacteroides acidifaciens*, *Heminiphilus faecis*, *Muribaculum intestinale*, *Candidatus Saccharibacteria bacterium*, *Duncaniella muricolitica*, and *Bifidobacterium pseudolongum* (Figures 4C, D). In the MX group, the top 10 most abundant genera at the genus level included *Bacteroides*, *Duncaniella*, *Eubacterium*, *Odoribacter*, *Acetatifactor*, *Desulfovibrio*, *Paramuribaculum*, *Muribaculum*, *Prevotella*, and *Alistipes*. At the species level, the most abundant species were *Ligilactobacillus murinus*, *Bacteroides acidifaciens*, *Paramuribaculum intestinale*, *Candidatus Saccharibacteria bacterium*, *Muribaculum intestinale*, and *Heminiphilus faecis* (Figures 4B,D).

LEfSe analysis was performed to identify significantly different species among the groups. It was found that the genus *Heminiphilus* was enriched in the ZG group, and at the species level, *Heminiphilus faecis* and *Paramuribaculum intestinale* were significantly enriched (Figure 4E).

Based on the gut microbiota analysis, this study reveals the microbial community characteristics following intervention with Huanglong Zhixiao Formula. The Venn diagram shows that Group ZG has a greater number of unique OTUs compared to Group MX, and its distinct composition of dominant genera and species at both taxonomic levels indicates that the formula can reshape the intestinal microecological structure in asthmatic model mice. Notably, LEfSe analysis identified the significantly enriched *Heminiphilus* genus and related species in Group ZG, which may serve as potential biomarkers of therapeutic efficacy. These findings provide new evidence for elucidating the mechanism by which Huanglong Zhixiao Formula exerts its therapeutic effects through modulation of the gut microbiota.

### 3.5 Fungal composition and differential fungal analysis

**Figure 5:**
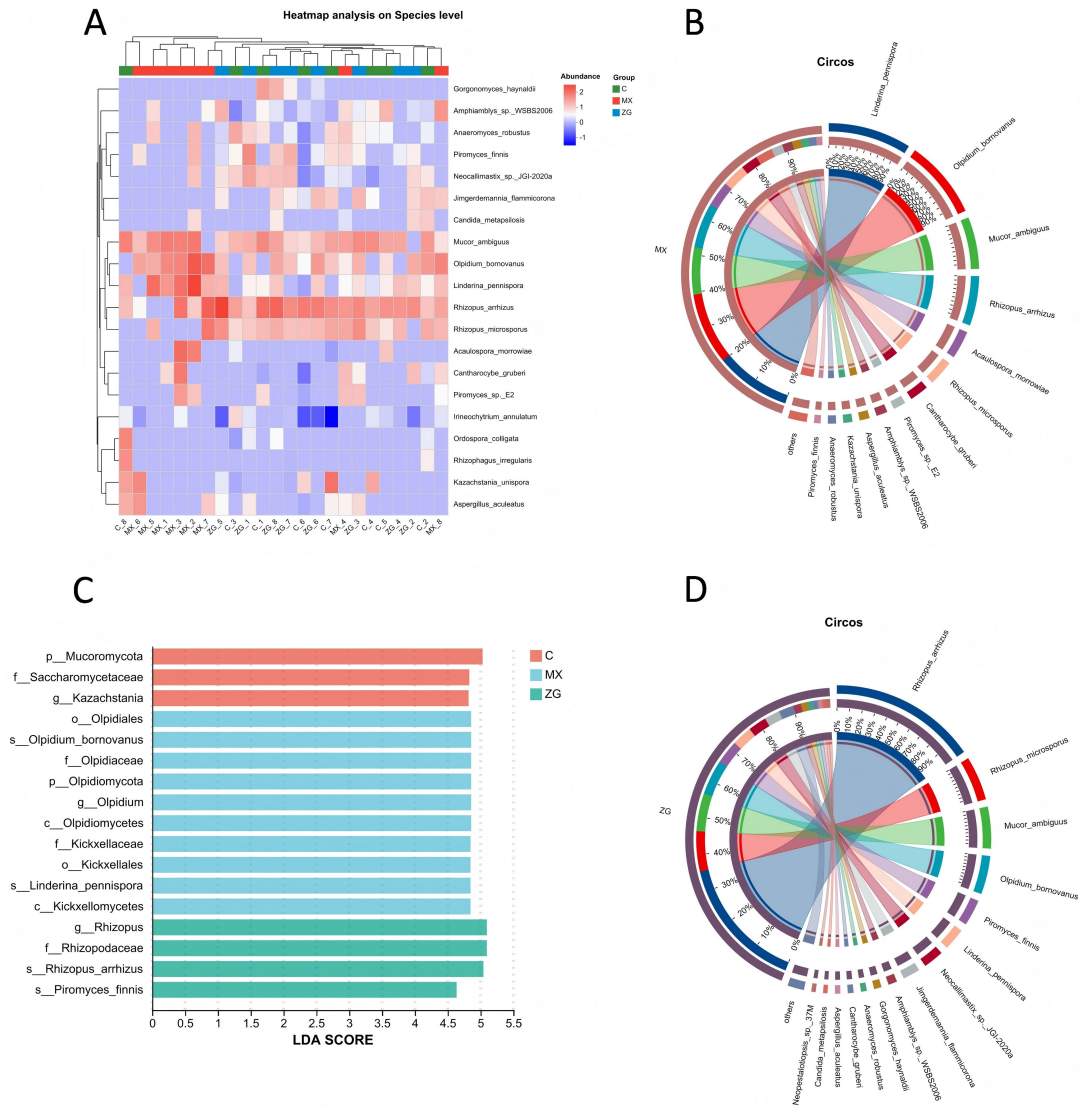
Fungal composition and differential fungal analysis in each group. **(A)** Heatmap analysis of gut fungal at the species level in each group (only the top 20 are shown); **(B,D)** Circos plots of gut fungal species at the species level in the MX/ZG groups (the left semicircle represents the species abundance composition of the samples, with different colors representing different species and the length corresponding to the abundance proportion; the right semicircle represents the distribution proportion of species in the samples, with colors representing the samples and the length corresponding to the proportion. The width of the connecting bands indicates the species abundance or distribution proportion, and the values outside the circle represent the species abundance); **(C)** Heatmap analysis of fungal at the species level in each group (only the top 20 are shown) (C: Control group, MX: Model group, ZG: HLZXF group).

Through sequence alignment analysis, the top 10 most abundant fungi detected at the species level in the MX group were *Linderina pennispora*, *Olpidium bornovanus*, *Mucor ambiguus*, *Rhizopus arrhizus*, *Acaulospora morrowiae*, *Rhizopus microsporus*, *Cantharocybe gruberi*, *Piromyces* sp. E*2*, *Amphiamblys* sp. WSBS*2006*, and *Aspergillus aculeatus* (Figures 5A,B). In the ZG group, the top 10 most abundant fungi detected at the species level were *Rhizopus arrhizus*, *Rhizopus microsporus*, *Mucor ambiguus*, *Olpidium bornovanus*, *Piromyces finnis*, *Linderina pennispora*, *Neocallimastix* sp. *JGI-2020a*, *Jimgerdemannia flammicorona*, *Amphiamblys* sp. *WSBS2006*, and *Gorgonomyces haynaldii* (Figures 5A,D).

Using LEfSe to screen for significantly different fungal taxa between groups, it was found that in the MX group, the genus *Olpidium* was significantly enriched, as well as the species *Linderina pennispora* and *Olpidium bornovanus*. In the ZG group, the genus *Rhizopus* was enriched, and at the species level, *Piromyces finnis* and *Rhizopus arrhizus* were significantly enriched (Figure 5C).

Based on the fungal analysis, this study elucidates the distinct modulatory effects of Huanglong Zhixiao Formula on the intestinal fungal community. The differential enrichment of specific fungal taxa between the MX and ZG groups, such as *Olpidium* and *Linderina pennispora* in the former and *Rhizopus* along with *Piromyces finnis* in the latter demonstrates that the formula induces a significant restructuring of the mycobiota structure. Notably, the marked enrichment of *Rhizopus arrhizus* and *Piromyces finnis* in the ZG group suggests their potential role as key biomarkers and functional effectors in the therapeutic process, possibly through involvement in polysaccharide degradation and metabolic regulation. These findings provide mycological evidence supporting the role of fungal community regulation in the mechanism of Huanglong Zhixiao Formula for asthma treatment.

### 3.6 Metabolic pathway prediction analysis

**Figure 6:**
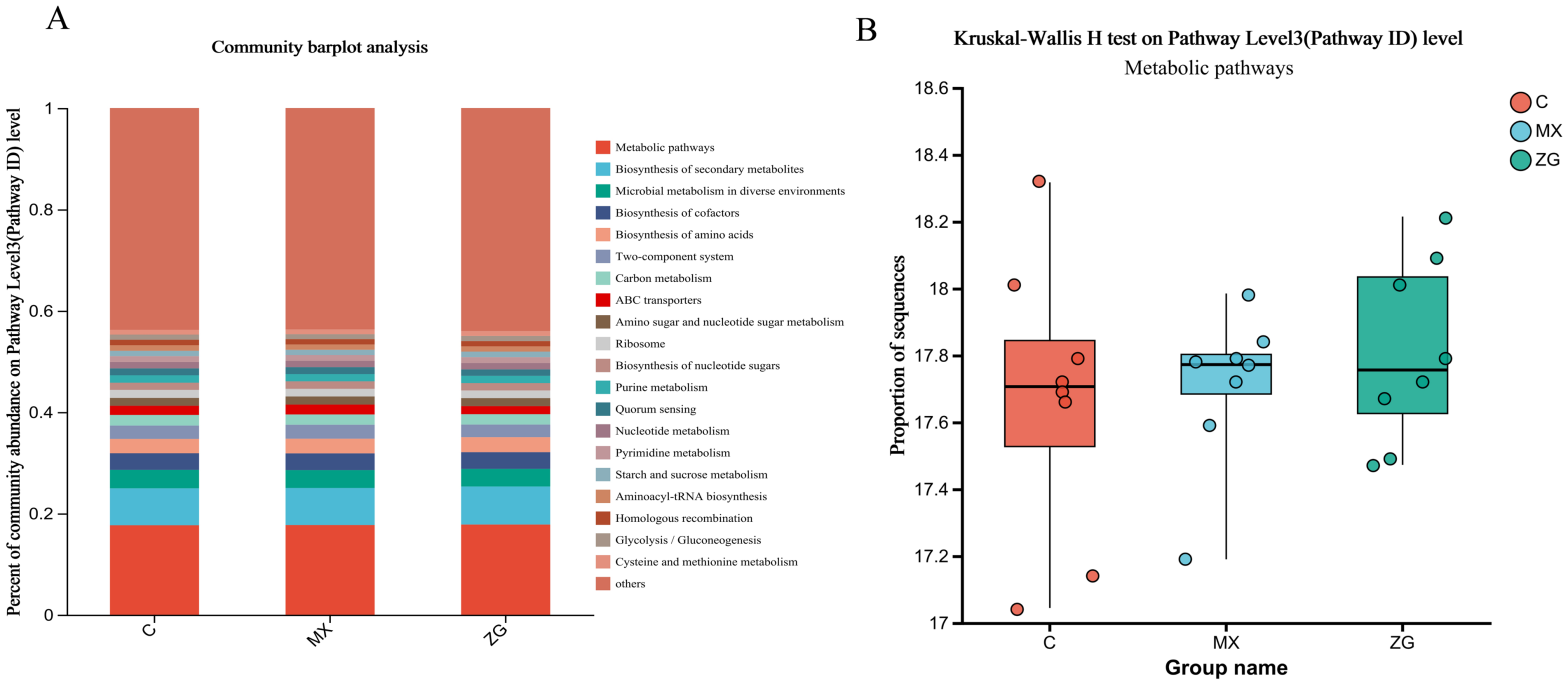
KEGG metabolic pathway prediction analysis. **(A)** Distribution of metabolic pathways involved in each group; **(B)** Differential prediction analysis of KEGG metabolic pathways among groups. (C: Control group, MX: Model group, ZG: HLZXF group).

Through the prediction analysis of metabolic pathways, no significant differences were found among the groups. The HLZXF group was mainly involved in the following pathways: Biosynthesis of secondary metabolites, Biosynthesis of cofactors, Biosynthesis of amino acids, as well as Microbial metabolism and Carbon metabolism (Figure 6).

### 3.7 The effects of HLZXF on intestinal and pulmonary pathology

**Figure 7:**
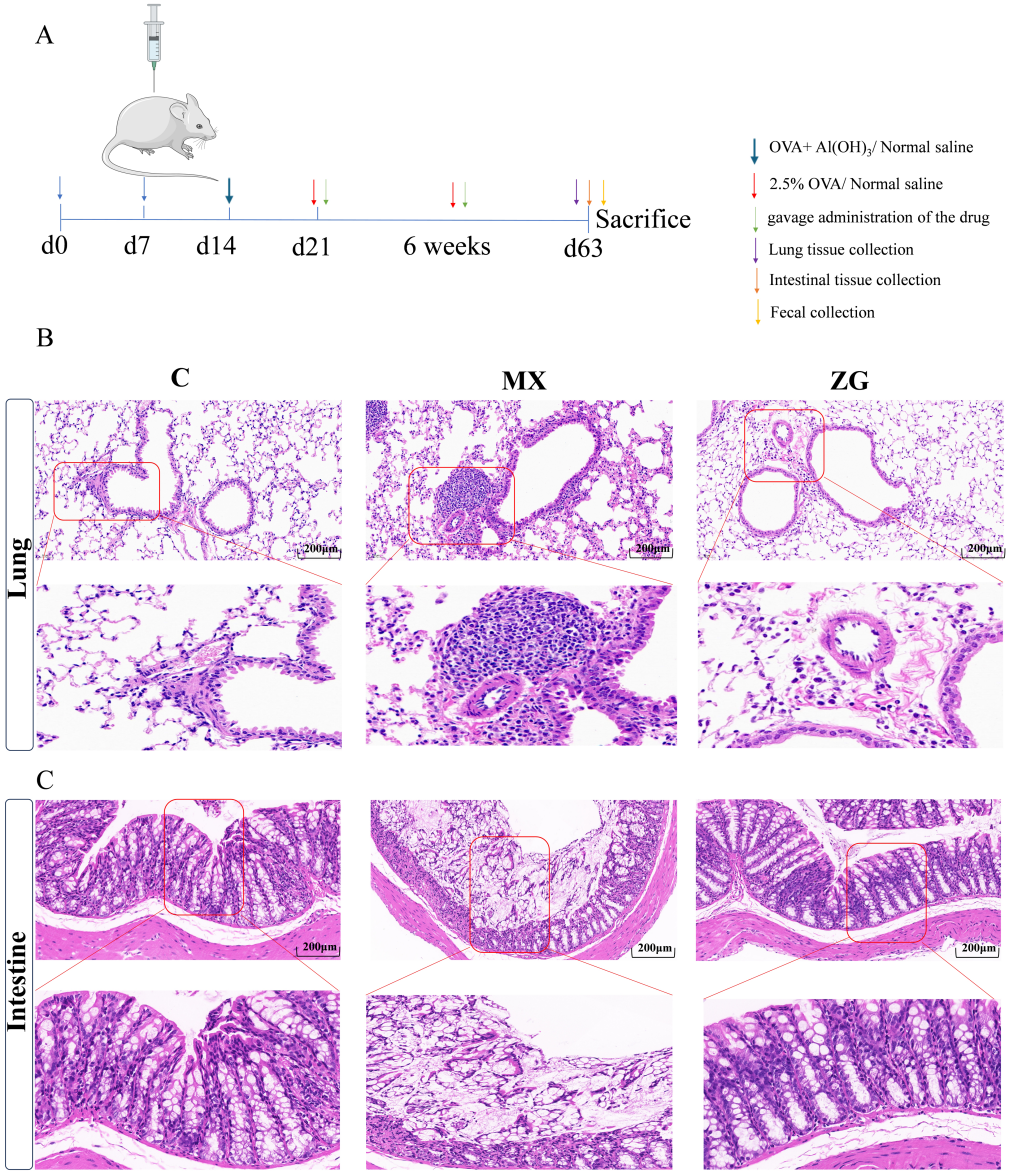
HLZXF treatment reduces pulmonary inflammation in asthmatic mice. **(A)** Schematic diagram of model construction and sample collection; **(B)** H&E staining of lung tissue (200X); **(C)** H&E staining of intestinal tissue (200X). (C: Control group, MX: Model group, ZG: HLZXF group).

To assess the effect of HLZXF on intestinal and pulmonary pathology, lung and intestinal tissues were collected from mice after asthma model establishment and HLZXF treatment (Figure 7A). Subsequently, the collected lung and intestinal tissues were processed for H&E staining, followed by pathological observation. Pathological observations showed that in the C group, the alveolar septa were thin and intact, no lung tissue damage or abnormal cellular infiltration was observed, and the pulmonary mucosal structure was normal. In contrast, in the MX group, the alveolar septa and tracheal walls were significantly thickened, the bronchial lumens were narrowed, and the lung tissue structure was disorganized. In the ZG group, the thickening of bronchial walls was significantly alleviated, no obvious structural deformities were observed, and the degree of alveolar rupture and fusion was relatively mild. This result indicates that HLZXF can effectively alleviate the damage to bronchial and alveolar wall structures in asthma model mice (Figure 7B).

In the C group, the intestinal tissue structure was normal, with no obvious pathological changes in intestinal villi or intestinal mucosa. However, in the MX group, the intestinal tissue structure was obviously disorganized, with lymphocyte and plasma cell infiltration in the lamina propria, fragmented intestinal epithelial cells, and a significant reduction in goblet cell numbers. After HLZXF treatment, only a small number of lymphocytes and plasma cells infiltrated the lamina propria; although the number of goblet cells was slightly reduced, the overall intestinal tissue structure remained intact (Figure 7C).

### 3.8 HLZXF promotes intestinal barrier repair by regulating protein expression

**Figure 8:**
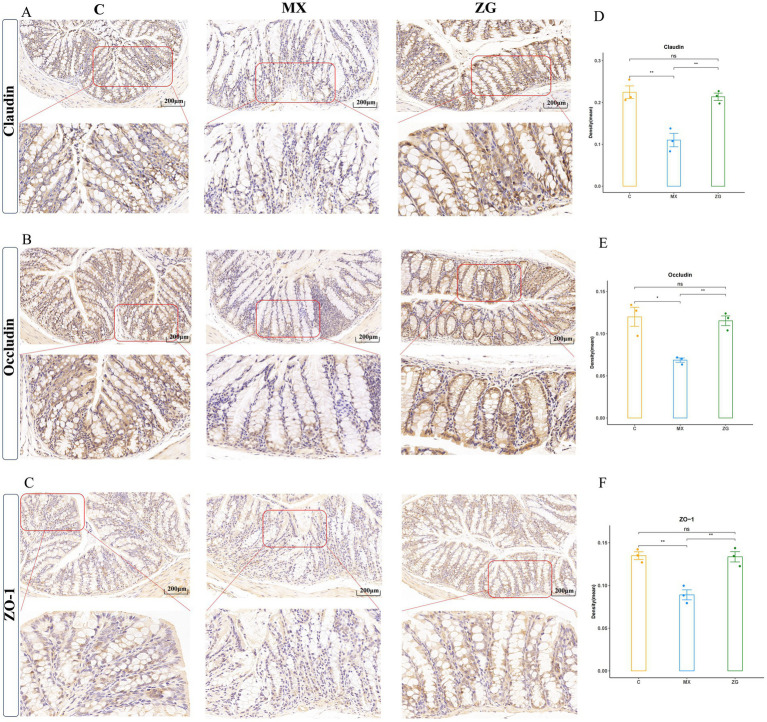
Immunohistochemical detection of the expression of Claudin, Occludin, and ZO-1 proteins in intestinal tissues. **(A)** Expression of Claudin protein (200X); **(B)** Expression of Occludin protein (200X); **(C)** Expression of ZO-1 protein (200X); **(D)** Statistical chart of Claudin protein Density (Mean); **(E)** Statistical chart of Occludin protein Density (Mean); **(F)** Statistical chart of ZO-1 protein Density (Mean). (C: Control group, MX: Model group, ZG: HLZXF group, *p < 0.05, **p < 0.01, ns: not significant).

To investigate the reparative effect of HLZXF on the damaged intestinal mucosal barrier, this study used IHC to detect the expression levels of Claudin, Occludin, and ZO-1 in mouse intestinal tissues. The results showed that, compared with the C group, the expression levels of Claudin, Occludin, and ZO-1 were significantly decreased in the MX group. However, after HLZXF intervention, the expression levels of Claudin, Occludin, and ZO-1 were significantly increased in the ZG group (Figure 8). In addition, when analyzing the inflammatory factors IL-22 and IL-33 in colon tissues, the relative expression levels of IL-22 and IL-33 in the MX group were significantly higher than those in the Group C (Supplementary Figure S1). This result demonstrates that HLZXF can effectively promote the repair of the damaged intestinal mucosal barrier in asthma model mice.

## 4 Discussion

This study investigated the regulatory effects of HLZXF on gut microbiota homeostasis in asthmatic mice and its underlying immune mechanisms, with the aim of providing new theoretical evidence for the treatment of asthma using TCM. The results showed that HLZXF significantly improved gut microbiota structure in asthmatic mice and alleviated asthma-related symptoms, which was achieved by modulating intestinal barrier function and the immune microenvironment.

In terms of gut microbiota diversity, the *α* diversity of gut microbiota in asthmatic model mice was significantly decreased, which is consistent with previous studies reporting reduced gut microbiota diversity in asthma patients (Alcazar et al., 2022; Abdel-Aziz et al., 2025: Liu et al., 2025). This finding suggests that gut microbiota dysbiosis may be one of the key factors contributing to the pathogenesis of asthma. However, in the ZG group, although gut microbiota diversity was not significantly changed, gut microbiota composition was significantly altered. Specifically, in the intestines of mice in the ZG group, the genera *Heminiphilus faecis* and *Paramuribaculum intestinale* were significantly enriched. *Heminiphilus faecis*, a Gram negative bacterium, is a beneficial microbe associated with the restoration of intestinal barrier function (Wang et al., 2025). *Paramuribaculum intestinale* is highly abundant in damaged tissues and is associated with tissue repair (Feng et al., 2022; Wei et al., 2022; Xia et al., 2023). Fungi also play an important role in the pathogenesis of asthma: gut-resident fungi can activate the immune system and induce the production of inflammatory factors. These inflammatory factors can be transported to the lungs via the bloodstream, where they modulate the pulmonary immune response, thereby triggering or exacerbating asthma related symptoms (Millien et al., 2013; Landers et al., 2019; Lu et al., 2024; Ding et al., 2025). In the present study, *Linderina pennispora* and *Olpidium bornovanus* were significantly enriched in the MX group, while *Piromyces finnis* and *Rhizopus arrhizus* were significantly enriched in the ZG group. To date, no published studies have reported the roles of these four fungal species in asthma. These findings suggest that HLZXF may restore gut microbiota balance by selectively modulating the abundance of specific bacterial and fungal populations, rather than simply increasing gut microbiota diversity, thereby exerting a beneficial effect on asthma.

HLZXF significantly alleviated intestinal and pulmonary pathological damage in asthmatic mice. H&E staining results showed that the lung and intestinal tissues of MX mice exhibited significant inflammatory cell infiltration and structural destruction. However, these pathological changes were significantly alleviated in the ZG group. Specifically, in the ZG group, the number of inflammatory cells in lung tissues was significantly reduced, and the structural destruction of bronchial walls and alveoli was partially reversed. In intestinal tissues of the ZG group, inflammatory cell infiltration in the lamina propria was reduced, and the integrity of the intestinal mucosal barrier was preserved. These results indicate that HLZXF not only directly alleviates pulmonary inflammation but also indirectly reduces it by modulating gut microbiota composition.

Further IHC detection showed that the expression levels of tight junction proteins (Claudin, Occludin, and ZO-1) in the intestinal tissues of MX mice were significantly downregulated, indicating impaired intestinal barrier function. However, in the ZG group, the expression levels of these proteins were significantly upregulated, IL-22 plays a crucial role in mucosal barrier function. Its receptor is expressed on epithelial cells, and it exerts a protective effect in patients with asthma by inhibiting the expression of pro-inflammatory chemokines and adhesion molecules (Wolk et al., 2004; Pennino et al., 2013). In our analysis, IL-22 was highly expressed in the model group. Consistent with our findings, high expression of IL-22 has also been reported in asthma models in other studies, which may be associated with the repair of the intestinal barrier (Besnard et al., 2011). IL-33 is a key early alarm factor in asthma. Released following airway epithelial injury, it activates the ST2 receptor, induces the production of IL-4, IL-5, and IL-13 by Th2 cells, mast cells, and other cell types, drives eosinophilic inflammation and airway hyperresponsiveness, and is closely linked to asthma susceptibility. Blockade of the IL-33/ST2 axis can alleviate airway inflammation. In the present study, IL-33 expression was also increased in the model group, while it was reduced in the intervention group treated with HLZXF (Schuijs et al., 2024; Asrat et al., 2025). suggesting that HLZXF can promote the repair of the damaged intestinal mucosal barrier. The restoration of intestinal barrier function is crucial for maintaining gut microbiota stability and may also alleviate pulmonary inflammation through gut lung axis signaling, thereby improving asthma related symptoms (Budden et al., 2017; Li et al., 2024).

While investigating the regulatory effects of HLZXF on gut microbiota and immune mechanisms in asthmatic mice, this study obtained certain findings but also had several limitations. First, this study only used BALB/c mice to establish the asthma model. Although this model can simulate certain pathological features of asthma, its uniform genetic background limits the ability to fully replicate the complex heterogeneity of human asthma. In addition, mouse sex can also affect asthma pathogenesis and severity. Several epidemiological studies have confirmed that asthma symptoms in females tend to worsen during specific age periods (e.g., post puberty and pre menopause) (Troisi et al., 1995; Real et al., 2008). Androgens (e.g., testosterone and dehydroepiandrosterone, DHEA) have been shown to reduce asthma incidence and may also alleviate asthma related symptoms (DeBoer et al., 2018; Chowdhury et al., 2021). Although this study implemented quality control measures and environmental standardization, it cannot completely rule out the potential impact of sex hormones on asthma outcomes. Second, although metagenomic sequencing identified changes in gut microbiota composition, this study did not perform *in vitro* experiments or microbiota transplantation to verify the causal relationship between specific microbial taxa and asthma improvement. It remains unclear whether the observed microbiota changes directly contribute to asthma symptom alleviation or if they are an indirect effect of other factors related to HLZXF intervention. In addition to the gut microbiota, the oral and nasopharyngeal microbiota as well as the virome also influence the pathogenesis of asthma (van Beveren et al., 2024; Zelasko et al., 2025; Berdnikovs et al., 2025). As important portals for the human body to interact with the external environment, the dynamic balance of the microbial communities and virome in the oral cavity and nasopharynx is closely associated with the occurrence and development of asthma. When this balance is disrupted, it leads to reduced microbial diversity, excessive proliferation of harmful bacteria, and the induction of local inflammatory responses (Bogaert et al., 2004; Dickson et al., 2014; Teo et al., 2015). The virome also plays a critical role in asthma pathogenesis. It can directly invade the epithelial cells of the nasopharyngeal and airway mucosa, causing cellular damage and impairing the integrity of the mucosal barrier, thereby rendering it more susceptible to allergens and pathogenic bacteria. Furthermore, viral infections can induce local immune dysregulation, enhance Th2 type immune responses, and promote IgE production. These effects further exacerbate chronic airway inflammation and drive the progression of asthma from intermittent episodes to a persistent disease course (Berdnikovs et al., 2025; Barra et al., 2025; Fahy et al., 2025). Furthermore, changes in the gut microbiota may represent a secondary response rather than a primary cause of asthma, although they may exacerbate host responses over the long term. In future studies, we will further consider expanding the sample size, introducing multiple animal models, and integrating in vitro experiments to verify the functions of specific microbial taxa. This will help elucidate whether a feedback loop exists between the gut and the lung—one that amplifies effects in both tissues over time—so as to more comprehensively reveal the mechanism of action of HLZXF.

Traditional asthma treatment primarily focuses on suppressing airway inflammation, alleviating airway hyperresponsiveness, and mitigating airway remodeling. However, recent studies have demonstrated that gut microbiota dysbiosis is closely associated with the pathogenesis of asthma. The results of this study indicate that HLZXF can significantly modify the composition and function of gut microbiota in asthmatic mice, enrich beneficial microbial taxa, and upregulate the expression of intestinal mucosal barrier related proteins. It further regulates the pulmonary immune microenvironment via the gut lung axis, thereby alleviating asthma related symptoms.

This finding suggests that in the future, TCM formulas could be integrated into comprehensive asthma treatment regimens, with gut microbiota modulation as the entry point, combined with existing anti inflammatory and bronchodilator therapies, to achieve a multi target and holistic treatment objective. Simultaneously, this study also provides a theoretical foundation for the development of novel asthma therapies targeting gut microbiota modulation, which may expand new directions for asthma treatment and improve patient clinical outcomes.

## Data Availability

The original contributions presented in the study are publicly available. This data can be found here: https://www.ncbi.nlm.nih.gov/, accession number PRJNA1377454.

## Supplementary material

SUPPLEMENTARY FIGURE S1 Analysis of inflammatory factor expression levels. **(A)** Analysis of IL-22 expression level; **(B)** Analysis of IL-33 expression level. (C: Control group, MX: Model group, ZG: HLZXF group, **p*<0.05, ns: not significant).
