# Supplementary figures and images for: Research on the regulation of gut microbiota homeostasis and immune function in asthmatic mice by Huanglong Zhixiao Formula
Source: Front Microbiol. 2026 Jan 5;16:1726388. doi: 10.3389/fmicb.2025.1726388 (PMC12813276; doi:10.3389/fmicb.2025.1726388)

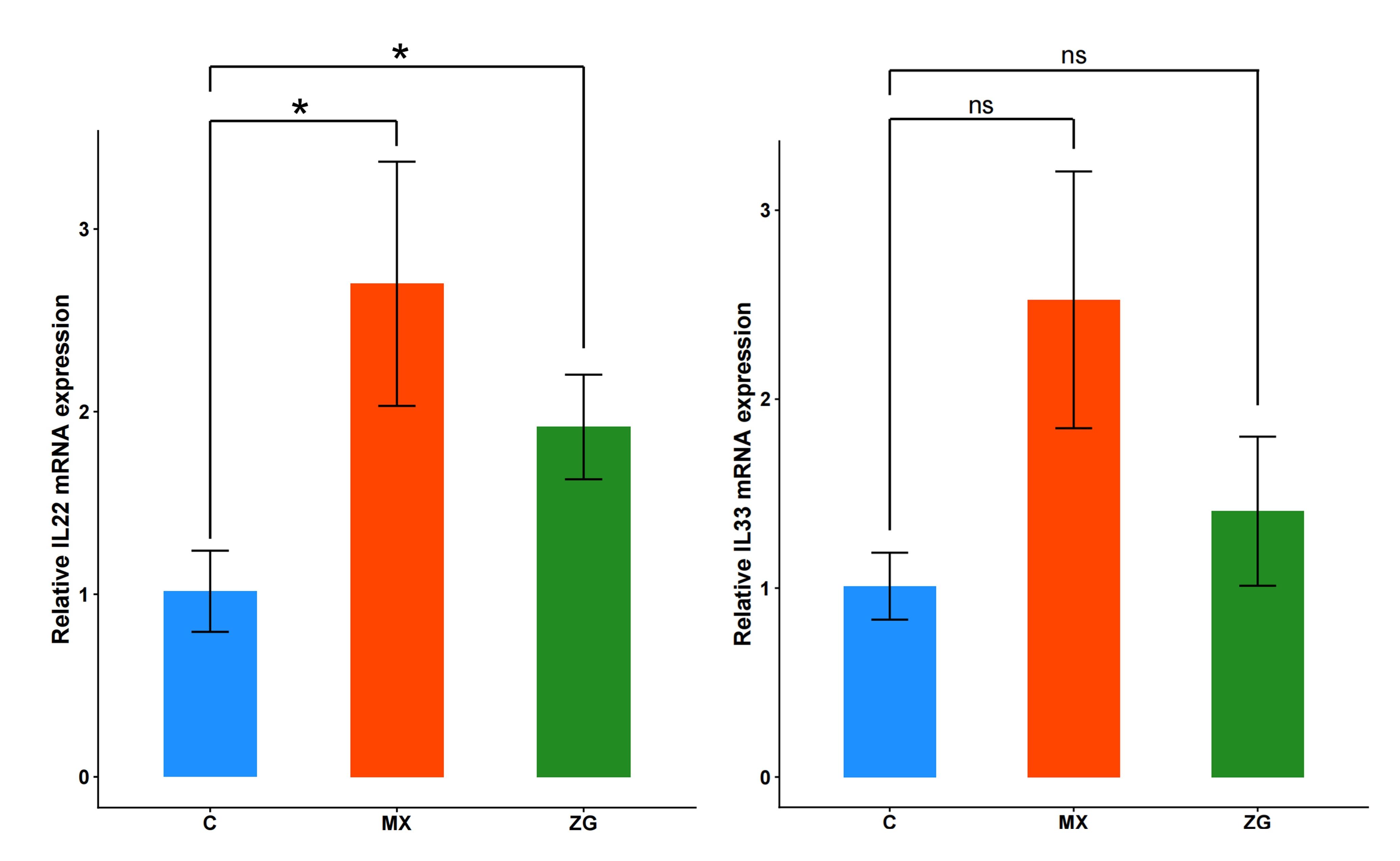

Supplement: SUPPLEMENTARY FIGURE S1 — Analysis of inflammatory factor expression levels. (A) Analysis of IL-22 expression level; (B) Analysis of IL-33 expression level. (C: Control group, MX: Model group, ZG: HLZXF group, *p<0.05, ns: not significant). [file Image_1.jpeg]
